# Cochlear Implants and Adult Patient Experiences, Adaptation and Challenges: A Survey

**DOI:** 10.3390/audiolres15060166

**Published:** 2025-11-30

**Authors:** Sahar Bin Dehaish, Abdulmalik Bin Marouq, Abdulaziz Almalki, Medhat Yousef, Fida Almuhawas, Abdulrahman Hagr, Jad Mony, Mohammad Albaqeyah, Hala Alferaih, Haifa Alqahtani, Sara Alghuraibi, Deepthi Poovayya, Hassan Yalcouy, Dalal Alrushaydan

**Affiliations:** 1 Cochlear Implant Centre, King Saud Medical City, Riyadh 12746, Saudi Arabia; sbindehaish@ksmc.med.sa (S.B.D.); abinmarzuq@ksmc.med.sa (A.B.M.); ab.almaleki@ksmc.med.sa (A.A.); 2 Audiology Unit, King Abdullah Ear Specialist Center (KAESC), King Saud University Medical City, Riyadh 12629, Saudi Arabia; myousef@ksu.edu.sa; 3 Department of Otolaryngology–Head and Neck Surgery, College of Medicine, King Saud University, Riyadh 11451, Saudi Arabia; fmuhawas@ksu.edu.sa (F.A.); hagr@ksu.edu.sa (A.H.); 4 King Abdullah Ear Specialist Center (KAESC), King Saud University Medical City, Riyadh 12629, Saudi Arabia; 5 Department of Audiology Unit, Cochlear & Bone Conduction Center, King Fahd Hospital, Jeddah 23325, Saudi Arabia; jmony@moh.gov.sa (J.M.); malbaqeyah@moh.gov.sa (M.A.); halfreeh@moh.gov.sa (H.A.); h.alqahtani1989@outlook.com (H.A.); alghuraybisarah@gmail.com (S.A.); 6 Cochlear Arabia Regional Headquarters, Riyadh 12361, Saudi Arabia; dpoovayya@cochlear.com (D.P.); hyalcouy@cochlear.com (H.Y.)

**Keywords:** cochlear implants, hearing loss, patient experience, access to care, age

## Abstract

Background: Cochlear implants (CIs) are a life-changing treatment for individuals with severe to profound hearing loss, yet adult CI uptake remains low despite high clinical and economic effectiveness. This study investigates adult patient experiences, adaptation, and barriers to CI access in Saudi Arabia. Methods: A survey of 89 adult CI recipients was conducted across three major CI centers in Saudi Arabia. The electronic questionnaire explored pre- and post-implant experiences, including referral pathways, device choice, adaptation, and satisfaction. Descriptive statistics, ranked correlations, and inferential tests were used to analyze associations between demographic and clinical variables. Results: The median time between hearing loss diagnosis and implantation was 17 years, with most patients using hearing aids beforehand. Healthcare professionals were the primary source of CI interest for 48% of respondents, though younger recipients were more often influenced by peers. Longer daily device use was linked to faster acclimatization (ρ = −0.26, *p* < 0.05); however, age, wait time, and initial attitude did not affect adaptation. Outcomes exceeded expectations for 54% of participants. Major barriers included lack of awareness (23%) and fear of surgery (18%). Only 4% learned about CI through social media. Advice for future candidates emphasized confidence and proactive action. Conclusions: Despite expanded CI availability in Saudi Arabia, structural and societal barriers persist. Empowering healthcare professionals and utilizing social media for awareness may enhance adult CI uptake and improve hearing health outcomes.

## 1. Introduction

Globally, over 1.5 billion people, more than 20% of the world’s population, are affected by some degree of hearing loss, of whom approximately 430 million experience disabling (moderate to profound) hearing loss requiring rehabilitation [1,2]. It is the third leading cause of Years Lived with Disability (YLDs) overall and the leading cause among individuals aged 70 and older [3]. In children, untreated hearing loss can delay speech and language development, contributing to reduced educational achievement, higher unemployment, and lower work productivity in adulthood [4,5]. Among older adults, it is linked to increased rates of depression and a higher risk of falls [6,7,8]. Globally, unaddressed hearing loss causes an economic burden of approximately $980 billion annually, according to the World Report on Hearing [9].

Given the significant individual and social impacts of untreated hearing loss, effective interventions are essential. For individuals with severe to profound hearing loss who derive limited benefit from conventional hearing aids, cochlear implants (CIs) represent a transformative solution [10]. According to Hoppe et al. (2023) and Zeitler et al. (2023), CI is likely to substantially improve hearing performance for individuals with insufficient speech perception using hearing aids and a hearing threshold of approximately 60 dB HL in at least one ear [10,11]. CIs have revolutionized auditory rehabilitation, offering life-changing benefits for those with significant hearing impairment [12]. These devices enable users to communicate and engage socially in ways that were previously unattainable [13].

The clinical and economic effectiveness of CI in adults and the elderly is well established, and there are more than one million CI users globally [14,15,16,17]. The World Health Organization identified CI as one of the most successful of all neural prostheses [3]. Nevertheless, still, only less than 5% who would benefit from a CI receive one [14,15]. Significant access barriers [16] contribute to the widespread undertreatment of adults with severe to profound hearing loss who could benefit from this life-changing intervention [4,18,19,20]. Identifying and addressing these barriers may improve the quality of life of approximately 40,000 Saudis, increase work productivity, support healthy aging and may create a substantial societal net benefit for the Kingdom of Saudi Arabia (KSA) [21,22,23].

While pediatric CI use exceeds 90% in some European countries, fewer than 5% of eligible adults receive the CI [24,25]. The main barriers are limited adult hearing screening and low awareness among healthcare providers and audiologists [26]. Neukam et al. (2024) reviewed 68 studies and categorized CI access barriers using an ecological model: (1) policy/structural (e.g., race, ethnicity, reimbursement), (2) societal (public awareness, education), (3) organizational (referral and geographic issues), (4) interpersonal (living situation, professional support), and (5) individual (surgical fears, hearing loss concerns, sound quality, and outcome uncertainty) [27].

Over the past decade, CI services in Saudi Arabia have advanced significantly. The implementation of nationwide neonatal hearing screening in 2016 marked a critical step forward in early detection [28]. Since then, early bilateral CI has become the standard of care for children with congenital or early-onset severe to profound hearing loss [29]. As of 2024, comprehensive reimbursement is available for bilateral CI in eligible children, adults, and older adults, including those with single-sided deafness [30]. Despite these substantial achievements, CI among adults and older individuals in Saudi Arabia remains limited and underutilized [30,31]. This study explores the real-world experiences, access barriers, and challenges faced by adults CI recipients in Saudi Arabia. By surveying CI recipients across three majors implant centers, we examine external factors like awareness, counseling, and referral pathways, alongside individual variables including initial attitudes, decision-making timelines, and post-implant experiences. This study addresses a critical gap in the literature by providing regional data on adult CI access in a country with expanding CI infrastructure and growing adult CI population, yet where access challenges and underutilization still persist. The findings aim to inform clinical practice, streamline referral strategies, and enhance public awareness to improve access and outcomes for adults CI candidates.

## 2. Materials and Methods

### 2.1. Data Collection and Questionnaire

The data collection for this survey was conducted between April and June 2024 across three major CI centers in Saudi Arabia: King Abdullah Ear Specialist Center (KAESC) in Riyadh, King Saud Medical City (KSMC) in Riyadh, and King Fahad General Hospital in Jeddah. The objective was to collect quantifiable demographic data and explore the experiences, attitudes, and challenges experienced by adult CI recipients at various stages of the implantation process. Participation in the survey was voluntary, and all respondents provided electronic informed consent before proceeding with the questionnaire.

A structured survey was developed by experienced audiologists specializing in CI. The survey was administered electronically via the “Qualtrics platform” (Qualtrics XM Platform, Provo, UT, USA), a cloud-based survey tool that operates on a continuously updated system without fixed version numbers) and included the following sections:

- Demographic data (age, gender, education, employment status);
- Pre-implantation experiences (duration of hearing loss, referral process, time to decision-making, and influencing factors);
- Surgical outcomes (device type, implant type, and subjective perspectives on outcomes);
- Post-implantation experiences (duration of use, psychosocial impact, and satisfaction with the implant).

The survey invitation was distributed via patient records maintained by the CI clinics. The clinics reviewed their records and applied primary inclusion criteria (current CI users and active patients at participating hospitals) to identify a pool of 382 eligible recipients. However, due to difficulties in immediately confirming the age criterion (Adults aged 18 years and above) for all potential participants based solely on the initial record list, the final eligibility screening and the application of exclusion criteria (including individuals below 18 years of age and data quality measures) were completed after the surveys were returned. After this final application of inclusion and exclusion criteria, 89 valid responses were included in the final analysis.

### 2.2. Sample Size and Sampling Method

A total of 382 CI recipients were invited to participate. The survey invitation was distributed via patient records maintained by the CI clinics, which reviewed their records and applied primary eligibility criteria (Current users of CIs and Active patients at participating hospitals) to identify the initial pool of invitees. Due to limitations in rapidly confirming the Adults aged 18 years and above criterion for all participants based on the initial record lists, the final eligibility screening and application of exclusion criteria were performed after the surveys were returned.

After applying all inclusion and exclusion criteria below, 89 valid responses were included in the final analysis. A random sampling approach was utilized to capture variations in age, gender, duration of implant use, and etiology of hearing loss.

Inclusion Criteria:

- Adults aged 18 years and above at the time of first CI.
- Current users of CIs.
- Active patients at one of the three participating hospitals.

Exclusion Criteria:

- Individuals below 18 years of age.
- Non-users of CI.
- Patients who were no longer receiving care from the participating hospitals.
- Survey forms with incomplete demographic information.
- Inconsistent responses across the survey.
- Unverified consent.

Data Quality Screening: To maintain data quality and reliability, responses were screened for accuracy and completeness. The final count of 89 valid responses used to calculate the 23.3% response rate strictly excludes surveys that contained incomplete demographic information, inconsistent responses, or unverified consent.

### 2.3. Statistical Analysis

Proportions, medians, interquartile ranges, and confidence intervals were used to summarize the survey data. Inferential statistics as applied to assumed relationships between demographic and outcome factors and included ranked analysis of variance (ANOVA), ranked correlation, and Chi-square tests.

### 2.4. Ethical Considerations

To maintain confidentiality and anonymity, data collection and analysis processes were carefully designed to prevent any identification of individual participants. Furthermore, participants were explicitly informed of their right to withdraw from the study at any point. This reliance on an anonymous, self-reported questionnaire meant that clinical variables could not be verified against institutional medical records.

## 3. Results

### 3.1. Patients

A total of 89 adults participated in the survey (demographic characteristics summarized in Table 1). The number of participants varies across characteristics because not all questions were mandatory. The onset of hearing loss, childhood, adult-fast, adult- progressive, and unknown was categorized based on respondents’ free text response to the questions such as: “What is the cause of your hearing loss? (if known)”.

### 3.2. Awareness and Access Barriers to CI

**Figure 1:**
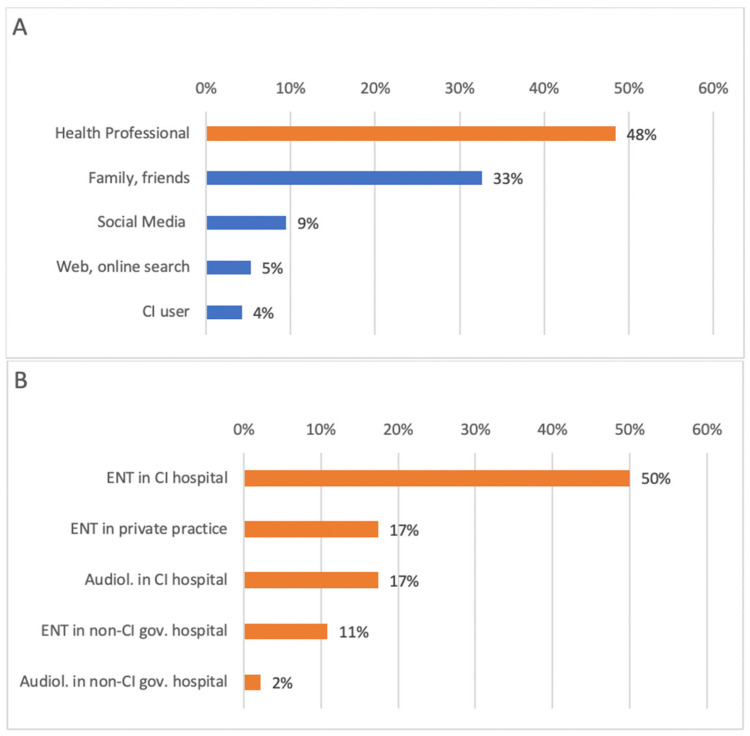
Distribution of primary that introduced respondents to CI treatment. (**A**): Primary sources of initial awareness about CI among participants. Orange bars represent healthcare professionals, while blue bars represent non-professional or public sources (family/friends, social media, web, or CI users) (*n* = 95). (**B**): Distribution of the types of healthcare professionals introducing CI to participants. Orange bars indicate different categories of ear, nose, and throat (ENT) specialists or audiologists, with darker shades distinguishing ENTs from audiologists. Percentages represent the proportion of respondents selecting each category (*n* = 46).

Interest about CI was triggered by health care professionals (HCPs) for nearly half (48%) of the respondents (Figure 1A). Figure 1A illustrates the distribution of the primary source that introduced respondents to CI treatment. The notation “Main source (*n* = 95)” indicates that 95 participants provided a response to this specific question regarding the primary source of their interest in CI. Notably, 67% of these professionals were employed at clinics that provide CI services (Figure 1B).

Age at implantation influenced the primary source of CI interest (*p* < 0.05, Cohen’s f = 0.25, *n* = 95). Younger recipients were more likely to learn from another CI recipient rather than from a physician (*p* < 0.001, Cohen’s d: 0.87, *n* = 50) or from family/friends (*p* < 0.05, Cohen’s d: 0.61, *n* = 35). Onset of hearing loss or years of CI use did not affect primary source of interest.

### 3.3. Candidate Attitudes Toward CI

**Figure 2:**
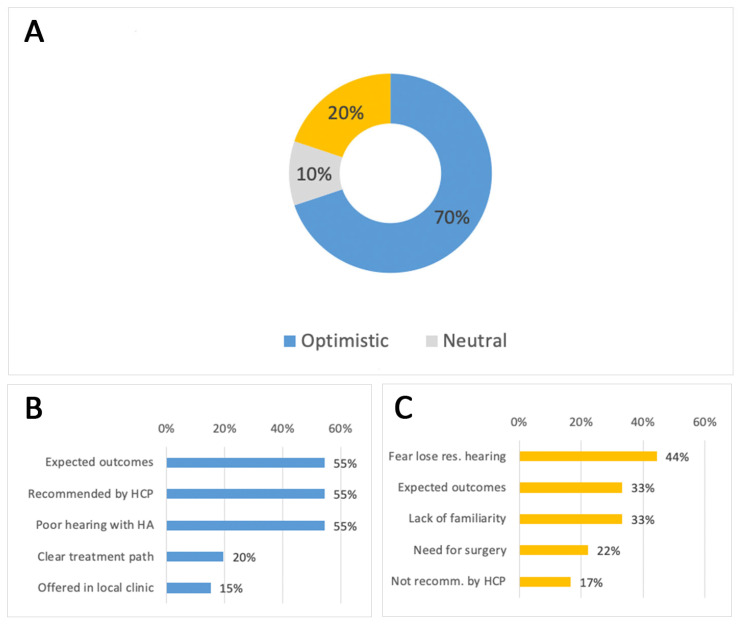
Initial Attitudes Toward CI at Candidacy Stage. (**A**). Distribution of respondents’ initial attitudes toward CI (*n* = 96). Blue represents optimistic respondents, gray represents neutral respondents, and yellow represents skeptical respondents. (**B**). Top five factors contributing to optimism (multiple responses allowed, *n* = 66). Blue bars indicate positive motivational factors influencing acceptance of CI (e.g., expected outcomes, healthcare professional recommendation, or poor hearing with hearing aids). (**C**). Top five factors contributing to skepticism (multiple responses allowed, *n* = 18). Yellow bars indicate concerns or negative factors contributing to hesitation toward CI (e.g., fear of losing residual hearing, uncertainty about outcomes, or surgical concerns). Abbreviations: HCP = Health Care Professional; Res = Residual; Recomm = Recommended.

When first learning about CIs, 67% of participants reported an optimistic attitude, while 11% were neutral, and 22% skeptical (Figure 2A). Optimism was mainly driven by (a) the expected, good hearing outcomes with a CI, (b) the fact that a healthcare professional recommended the treatment and (c) the limitations experienced with using hearing aids (Figure 2B). In contrast, skepticism mainly stemmed from (a) fear of losing residual hearing, (b) concerns about expected outcomes after CI, and (c) lack of familiarity with CIs (Figure 2C).

A moderate association was found between the primary source of CI interest and initial attitude (Cramer’s V = 0.31; χ^2^ (df = 8, *n* = 94) = 17.7, *p* < 0.05). Recipients with an initially optimistic attitude were most likely to have learned about CIs from other CI users, while those who were initially sceptical were more often informed by family or friends. Initial attitude was not influenced by hearing loss onset, age at implantation, or duration of hearing aid use.

### 3.4. Delays on the Path to Treatment

**Figure 3:**
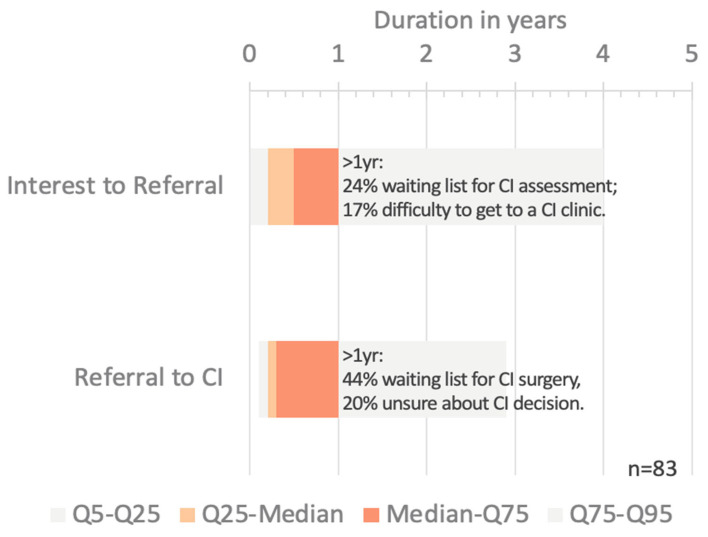
Time period between initial interest in CI treatment and appointment with a CI specialist (*n* = 90), and between first CI specialist appointment and surgery (*n* = 88). Bars represent the distribution of durations between stages. Time intervals are divided into quantiles as follows: Q5–Q25: 5th to 25th percentile (early cases); Q25–Median: 25th to 50th percentile; Median–Q75: 50th to 75th percentile; Q75–Q95: 75th to 95th percentile (later cases). Each segment reflects the proportion of individuals falling within that time range, illustrating variability across the population.

Participants reported a median period of 17 years (IQR: 2–23 years) between hearing loss diagnosis and first CI. Most (85%, *n* = 86) had used hearing aids prior to implantation, with a median use of 15 years (IQR: 5–23 years) (Figure 3). Participants reported a median delay of 17 years (IQR: 2–23 years) between the diagnosis of hearing loss and receiving their first CI, despite most (85%, *n* = 86) having used hearing aids for a median duration of 15 years (IQR: 5–23 years). Notably, the longest delays were observed prior to referral and engagement with a CI center, aligning with the observation that once patients reach a CI specialist, the process tends to move more efficiently. After expressing interest in CI, the median time to a specialist appointment was only 0.5 years (IQR: 0.2–1 year), followed by a median wait of 0.3 years (IQR: 0.2–1 year) for surgery. For those experiencing delays over one year, the most common barriers were clinic waiting lists (25–42%) and travel difficulties (19%), along with uncertainty around candidacy (21%), highlighting the need to streamline referral processes and improve access earlier in the care pathway (Figure 3).

**Figure 4:**
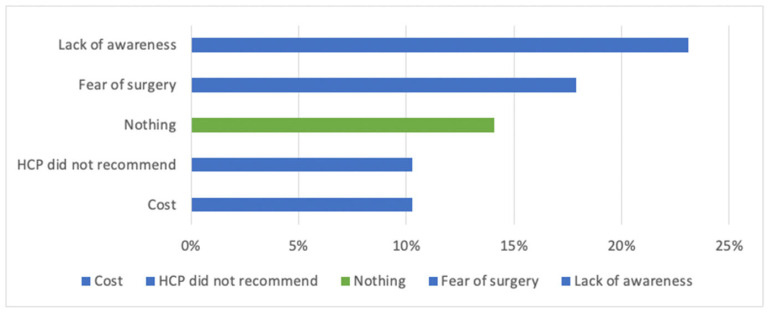
Self-reported barriers to earlier CI adoption. Top five self-stated major barriers to earlier adoption of CI. (*n* = 78).

CI candidates with a shorter time between initial interest and their first CI-specialist appointment were also more likely to proceed quickly from that appointment to surgery (*p* < 0.001, Spearman’s ρ = 0.50, CI: [0.32–0.64], *n* = 86). Older age at implantation was associated with faster progression from initial interest to surgery (*p* < 0.05, Spearman’s ρ = –0.22, CI: [–0.41 to –0.01], *n* = 86). Hearing loss onset, primary source of interest, CI experience duration, and initial attitude toward CI had no significant effect on treatment timing. In free-text responses, participants described what had initially delayed them from pursuing CI. The most common reasons were (a) lack of awareness (23%) and (b) fear of surgery (18%). Figure 4 presents the top five response categories (Figure 4).

Participants with shorter-than-median wait times from initial CI interest to treatment were moderately more likely to report lack of awareness as a barrier (χ^2^ (df = 1, *n* = 74) = 8.1, Cramer’s V = 0.33, *p* < 0.05), and moderately less likely to report treatment cost as a barrier (χ^2^ (df = 1, *n* = 74) = 8.5, Cramer’s V = 0.34, *p* < 0.05). Perceived barriers to earlier treatment were not influenced by hearing loss onset, age at implantation, or CI experience duration.

### 3.5. Device Choice

Before CI, participants selected an implant and sound processor type. The majority (67%) reported choosing based on their CI specialist’s recommendation. Additionally, 32% were influenced by other CI users, and 30% by specific device features. Notably, 25% (*n* = 92) indicated they had no option to choose a device (multiple responses possible).

### 3.6. Post-Implantation Perspectives

**Figure 5:**
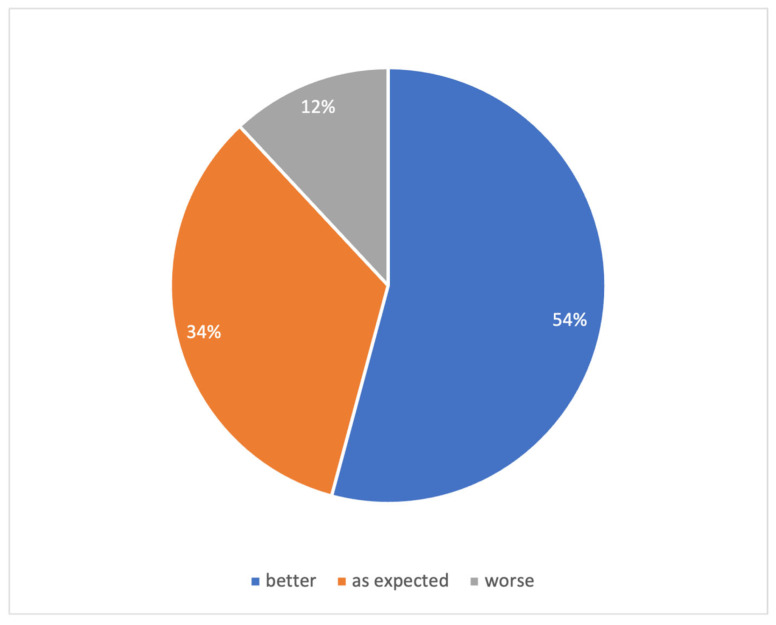
Post-implantation outcomes compared to pre-implant expectation. Participants’ self-assessed outcomes after CI use, categorized by whether expectations were exceeded, met, or not met (*n* = 90).

Respondents (*n* = 89) typically reported needing four months to adjust to their CI (IQR: 2.4–12 months). The median daily use was 12 h (IQR: 9–15 h). Longer daily use was associated with faster acclimatization (*p* < 0.05, Spearman’s ρ = –0.26, CI: [–0.45; –0.05], *n* = 83). Time to adjust was not related to age at implantation, wait time from initial interest in surgery, hearing loss onset, or initial attitude toward CI. In retrospect, most respondents (54%) reported that CI outcomes exceeded their expectations. For 33%, outcomes met expectations, while 12% reported outcomes were below expectations (Figure 5).

Reported outcomes relative to pre-treatment expectations were not associated with daily device use, age at implantation, time from initial interest to treatment, initial attitude toward CI, hearing loss onset, or time needed to adjust to the CI. At the end of the questionnaire, respondents (*n* = 78) shared practical advice for individuals newly considering CIs. Most responses fell into the following categories: (a) “Be more confident” (59%), (b) “Act quickly” (35%), and (c) “Seek counselling” (15%) (Table 1).

## 4. Discussion

The findings of this study provide an understanding of the journey adults undergo with CI, including barriers to access, factors influencing decision-making, and post-implantation outcomes. A key insight is the significant gap in awareness about CI, both among potential candidates and healthcare providers. This aligns with previous studies, such as those by Yang et al. 2017 [32] and Dettman et al., 2016, [33], which identified similar deficiencies in education and outreach as barriers to treatment adoption. Fear of surgery, socioeconomic challenges, and concerns about residual hearing loss were also cited, consistent with findings from prior research [27]. These parallels strengthen the need for comprehensive, targeted interventions to address these challenges.

Many of the adult CI access barriers described previously also appeared in this Saudi Arabia specific study. The finding that the median time between diagnosis and implantation was 17 years, while the process moves relatively quickly once the patient is referred to a CI specialist (median 0.5 years from interest to appointment), confirms that the primary impediment occurs during the patient’s initial journey through general healthcare setting. In line with Buchman et al. (2020) and Neukam et al. (2024), the lack of awareness amongst HCPs outside CI centres—resulting in a lack of referring to CI assessment, appears as the main contributor to the long period from hearing loss diagnosis to CI treatment [12,27]. As CI is typically only done in central, specialized hospitals, a transparent referral path from community or primary hearing care clinics to CI providing hospitals is essential [34]. For this to happen, hearing and other HCPs need to be empowered to identify CI candidates and to motivate them to undergo CI assessment [35]. Interestingly, in the present study, nearly half of the participants reported interest about the CI was triggered by HCPs (48%), which reinforces the crucial role of HCPs in disseminating knowledge related to CI assessment. However, younger individuals were more likely to learn about CI from another CI recipient than from a physician (*p* < 0.001, Cohen’s d = 0.87, *n* = 50) or from family and friends (*p* < 0.05, Cohen’s d = 0.61, *n* = 35), likely because younger patients tend to be more receptive to information from diverse sources. A broader understanding of the secondary health consequence of untreated hearing loss, the effectiveness of hearing treatment and easy to apply guidelines with screening procedures and clear biomarker cut-off for referral are promising approaches to address this access barrier to adult hearing care.

In addition, our survey showed that older age at implantation was significantly associated with faster progression from initial interest to surgery. This may be due to older individuals having fewer competing responsibilities, such as work or caregiving, allowing them to prioritize hearing restoration more readily than younger adults. Furthermore, participants with shorter-than-median wait times were more likely to cite “lack of awareness” as a barrier, and less likely to report treatment cost as an obstacle. This suggests that improving early awareness may help shorten the overall pathway to CI, especially for motivated candidates with fewer structural limitations.

In our survey, 67% of participants expressed optimism when they first learned about CI, primarily driven by the fact that the treatment was recommended by a healthcare professional. In contrast, scepticism was largely rooted in fears of losing residual hearing and uncertainty about potential outcomes, likely influenced by a lack of awareness and insufficient reassurance from healthcare providers. Participants typically required around four months (IQR: 2.4–12 months) to adjust to their CI, with a median daily use of 12 h (IQR: 9–15 h). Notably, longer daily device use was moderately associated with faster acclimatization, emphasising the importance of consistent usage in early recovery. However, self-reported outcomes relative to expectations were not significantly influenced by daily device use, age at implantation, initial attitude, time from interest to surgery, or hearing loss onset. This indicates that while device adherence supports faster adaptation, subjective satisfaction may be influenced by broader psychological and counselling factors.

A low level of public awareness appears as another access barrier to CI assessment in Saudi Arabia. Only a small proportion of responders identified social media or the internet as source of interest in CI. A review by El Keir et al. (2021) identified social media as a cost-effective tool for addressing key health behaviour [36]. In the KSA, more than half of the surveyed public reported an interest in using social media for health-related purposes [37]. The review highlights the effective social media activities of the Ministry of Health, e.g., with infographics and videos improving health literacy amongst millions of followers, particularly amongst those with chronic health conditions [38]. Credible social media channels may allow existing CI users to effectively share their experience with CI candidates. Our data suggests that this currently underused channel (only 4%) may become a reliable way of awareness creation (all four responders who learned from a CI users were initially positive about CI [39].

Compared to the more than a decade between hearing loss diagnosis and CI treatment, the time from being interested in CI treatment to seeing a CI specialist, and the time from the first CI specialist appointment to treatment seems acceptable for most CI recipients [39]. In recent years increasing number of clinics in Saudi Arabia providing CI treatment and the introduction of updated indication criteria might already address barriers reported by CI recipients with a particularly long time between becoming interested in CI to treatment [40].

In our survey, relative to structural barriers, individual barriers such as fear of surgery seem to have played a relatively minor role. However, since we only surveyed actual CI recipients, it is unknown for how many CI candidates such individual barriers appear challenging. These findings align with the free-text advice offered by participants: the majority emphasized the importance of taking control of the decision-making process. The top three themes were “Be more confident” (59%), “Act quickly” (35%), and “Seek counselling” (15%). Together with the quantitative data, these insights highlight how empowerment, timely action, and professional guidance can shape more successful CI journeys. Fabie et al. (2024) measured CI candidate expectations with the CIQOL-36 before and after CI evaluation and before surgery [41]. They showed that counselling during the CI-evaluation increased expectations of those with initially poor expectations and decreased expectations of those with initially high expectations. In our study group, more than half of the respondents reported outcomes that exceeded their expectations, while only a small minority felt that the results fell short of what they had anticipated before surgery. Dillon et al. (2019) describe how CI candidates weigh up risks and benefits in the context of their own life and believes. This process may be well supported by health professional counselling and by patient interaction with peers [42].

In the context of Saudi Arabia, the cost of cochlear implantation, including surgery, device, and postoperative rehabilitation is covered by the public healthcare system for all eligible patients, thereby minimizing individual financial burden [40]. Nonetheless, each CI procedure represents a significant investment for the healthcare system, with international cost estimates ranging between USD 20,000 and 40,000 per implantation, depending on device and care components [43,44]. Importantly, multiple studies have demonstrated that cochlear implantation is highly cost-effective, yielding long-term quality-of-life gains and economic benefits that outweigh initial costs [43,45,46].

Although this study was conducted within the Saudi Arabian healthcare context, many of the findings such as delayed referrals, limited awareness among non-CI healthcare professionals, and the underutilization of cochlear implants in adults mirror global trends reported in studies from Europe, Australia, and North America [47]. These similarities suggest that the barriers identified are not unique to Saudi Arabia but reflect broader systemic challenges in adult hearing healthcare [47]. Nonetheless, some results are context-specific, as Saudi Arabia’s comprehensive public healthcare system fully covers the cost of CI surgery and rehabilitation, thereby minimizing financial barriers that remain significant in many other countries [48]. Consequently, while the conclusions regarding the need for improved awareness, referral pathways, and public education are widely generalizable, the economic and policy implications may differ across healthcare systems depending on funding models and service organization [49].

The findings should also be interpreted in light of characteristics specific to the Saudi Arabian healthcare system and cultural context. CI services are primarily centralized in tertiary hospitals, ensuring high-quality care but potentially prolonging referral timelines [30]. Although earlier only a few centers were available, the recent expansion of CI programs across the country has improved accessibility. Still, patients tend to prefer treatment at the nearest center, emphasizing the need for continued regional service development. Universal public coverage minimizes financial barriers, but cultural factors such as strong family involvement in medical decisions and perceptions surrounding hearing loss may influence the timing of seeking treatment. These contextual elements help explain the prolonged pre-referral intervals observed and highlight the importance of decentralized referral systems and public awareness initiatives.

### Strengths and Limitations

This study has several strengths. First, it provides a firsthand analysis of the experiences of adults with CI in the Kingdom of Saudi Arabia. The application of both qualitative and quantitative techniques facilitated a comprehensive understanding of participants’ experiences. This included patients’ post-implant perspective, enhanced by the benefit of hindsight. Combining the information from this Saudi patient survey with global trends reported in the literature, created actionable insights into how patient access to adult CI may be improved in the KSA

However, this study is not without limitations. First, although the structured survey was developed by experienced audiologists specializing in cochlear implants (CIs) and provided a comprehensive understanding of participants’ experiences using both qualitative and quantitative approaches, the instrument was not formally psychometrically validated (e.g., through reliability testing or factor analysis). This limits the internal reliability of the measures compared to established scales. Second, the study relied exclusively on anonymous, self-reported data, which, while ensuring participant confidentiality, prevented verification of certain clinical details (such as onset and progression of hearing loss) against medical records. This limitation contributed to a higher proportion of “unknown” responses for some variables (e.g., 37% for onset of hearing loss). Third, as participation in the survey was voluntary, the study may be subject to selection bias, since individuals with more positive attitudes toward CI or higher engagement in their care may have been more likely to respond. Furthermore, the survey targeted only existing CI recipients rather than including individuals who were evaluated but did not proceed with implantation, potentially omitting perspectives from those who were skeptical or declined treatment. Finally, the study did not explore whether discontinuation of care at a CI center corresponds to discontinuation of device use, as all participants were current CI users. Future studies should examine how follow-up continuity influences long-term CI use.

## 5. Conclusions

In conclusion, the study emphasizes the role of societal and structural healthcare access barriers, such as lack of health professional awareness and embedded referral paths, for adults with severe and higher-level hearing loss. Promising actions to reduce access barriers include empowering healthcare professionals to identify and refer CI candidates by implementing easy-to-follow guidelines for hearing screening and referral. Enhancing public awareness through credible social media activities may be a complementary strategy.

## Figures and Tables

**Table 1:** Characteristics of the individuals included in the study.

Characteristic		
Current Age (*n* = 86)	Median: 38 years	IQR: 27 to 46 years
Age at CI (*n* = 86)	Median: 30 years	IQR: 25 to 40 years
Duration of CI experience (*n* = 86)	Median: 3 years	IQR: 1 to 6 years
Hearing Solution (*n* = 86)	CI Unilateral	57%
	Bimodal	17%
	CI Bilateral	26%
Onset of hearing loss (*n* = 98) (Categorised from self-stated free text)	Childhood	29%
	Adult, fast onset	27%
	Adult, gradual onset	17%
	Unknown	37%
Education Level (*n* = 86)	Less than high school	21%
	High school/GED	21%
	College (2-year)	17%
	Bachelor’s degree	35%
	Master’s degree	5%
	Ph.D.	1%

## Data Availability

The data used and/or analyzed in this study is available and will be provided by the corresponding author upon request.

## Abbreviations

ANOVA	Analysis of Variance
APC	Article Processing Charge
CI	Cochlear Implant
CIQOL-36	Cochlear Implant Quality of Life-36
dB HL	Decibels Hearing Level
ERKI	*Erfassung des Richtungshörens bei Kindern* (Localization Testing in Children)
HCP	Health Care Professional
ILD	Interaural Level Difference
IRB	Institutional Review Board
IQR	Interquartile Range
KACST	King Abdulaziz City for Science and Technology
KSA	Kingdom of Saudi Arabia
LED	Light-Emitting Diode
LT	Left (ear)
ORCID	Open Researcher and Contributor ID
RT	Right (ear)
SD	Standard Deviation
SPL	Sound Pressure Level
SPSS	Statistical Package for the Social Sciences
